# Welfare Implications for Tigers in Travelling Circuses

**DOI:** 10.3390/ani14071053

**Published:** 2024-03-29

**Authors:** Emily Davies, Andrew Knight

**Affiliations:** 1 Representing Animals, 147 Station Road, London E4 6AG, UK; 2 School of Environment and Science, Nathan Campus, Griffith University, 170 Kessels Rd, Nathan, Brisbane, QLD 4111, Australia; 3 Faculty of Health and Wellbeing, University of Winchester, Sparkford Road, Winchester SO22 4NR, UK

**Keywords:** animal welfare, circus, five domains, tiger, *Panthera tigris*

## Abstract

**Simple Summary:**

Many countries continue to allow the use of non-domesticated animals, such as tigers, in travelling circuses, as introducing legislation or bans often requires sufficient scientific evidence that the environment negatively impacts animal welfare. Whilst we know that larger territorial animals are least suited to captive environments, to date there has been very limited investigation into the welfare of tigers in travelling circuses. By reviewing the scientific evidence available on the topic, this paper suggests that the travelling nature of a circus often negatively impacts on the suitability of the physical environment for tigers, as well as their nutrition, health, and mental state. However, training for performances could positively impact welfare, dependent on the techniques used. Nevertheless, the preponderance of the scientific literature supports additional nationwide bans on the use of tigers in travelling circuses internationally, due to animal welfare concerns.

**Abstract:**

There are very few studies that have focused on species-specific welfare implications for tigers in a travelling circus. The absence of scientific evidence to inform nationwide legislation means that tigers are still commonly used in travelling circuses across the world. A systematic review of relevant published studies was conducted using the bibliographic databases Web of Science and Scopus, supplemented by a narrative search. In total, 42 relevant studies were identified that assessed the welfare of tigers in captivity, including circuses and zoos. Only eight papers assessed the welfare implications for tigers in circuses directly, evidencing the lack of research in this area. Given that circuses provide a sub-optimal environment compared to zoos, implications for tiger welfare were also inferred from zoo research, within the Five Domains framework. Collectively, these papers infer that the travelling nature of a circus often negatively impacts the welfare domains of nutrition, physical environment, health, and mental state. This is due to limitations in enclosure size, as well as in both environmental and behavioural enrichment. There is also often difficulty in sourcing appropriate food and specialised routine veterinary care. The literature is divided concerning behavioural interactions, specifically whether training can improve welfare by offering mental stimulation. However, circus performances are often associated with negative welfare due to noise disruption from spectators. The collective scientific evidence indicates that tigers are not well suited to circus living, due to the inability of a travelling circus to provide for their species-specific psychological, physiological, and behavioural needs.

## 1. Introduction

### 1.1. Animal Welfare

Since animal welfare became an area of scientific interest around 60 years ago, the field has developed rapidly, with concepts and methods for assessing welfare continuing to be refined [1,2]. Broom ([3], p. 5) defined animal welfare as “the state of an animal as regards its attempts to cope with its environment”. The state will differ between individuals based on their subjective experiences [2,4,5]. Animal welfare includes physical, behavioural, and psychological components, can be positive or negative, and can change dependant on the individual’s experiences at a given time. The welfare of animals within various settings and circumstances can be inferred from the scientific literature, which is used to inform policy and create species-specific recommendations, such as the Association of Zoos and Aquariums (AZA) tiger (*Panthera tigris*) care manual [6].

In 1994, Mellor and Reid [7] published the Five Domains Model for animal welfare assessment. This has since been regularly updated to include the latest developments in the field [1,8,9,10,11,12,13]. This framework is increasingly being used within animal welfare legal cases and to inform policy [14].

The purpose of the domains is to draw attention to areas that are considered important for both animal welfare and management, enabling assessment of both positive and negative states [13]. In line with the most recent model, the Five Domains are: nutrition, physical environment, health, behavioural interactions, and mental state [1].

Negative or compromised animal welfare is determined when welfare indicators show that an animal is failing to cope, or has difficulty in coping, with its environment and often occurs with suffering [2,15]. Therefore, if welfare in any of these domains is substantially compromised, it can usually be inferred that the environment is harmful and thus not suitable for the animal.

### 1.2. The Welfare of Tigers within Circuses

As of June 2023, 52 countries had introduced bans or restrictions on the keeping of wild animals in circuses [16]. Many of these bans cite animal protection or animal welfare as their rationale [17]. However, some bans are species-specific and do not apply to all non-domestic animals [16]. Circuses have a limited ability to make improvements for animal welfare, due to restrictions in space and environmental enrichment, and their travelling nature, which necessitates frequent transportation [18].

Non-domesticated species, including tigers, appear least suited to a circus life [19]. However, scientific literature on the welfare of tigers in circuses is scarce, meaning that there is limited data to inform legislative decisions on policy [18]. A report arranged by the United Kingdom (UK) government’s Department for Environment, Food and Rural Affairs (DEFRA) [20] concluded that there was not sufficient scientific evidence to determine the welfare state of wild animals in travelling circuses, with members of the sub-groups failing to agree on whether performances, training, and transport have a positive or negative impact on the welfare of an animal. Research about circuses often investigates single issues such as enclosure size [21], transport [22], and training [23], without considering the overall welfare of the animals. Such research has often been conducted in larger, better financed circuses, and so likely also represents the best instances of husbandry and welfare in such establishments [19].

Holistic reviews on the welfare of animals in circuses often make inferences from research conducted in zoos [19,24]. The captive environments are similar in terms of imposing limitations on the physical environment, diet, access to healthcare, and enforced social interactions. However, whilst many zoos are guided by species-specific care manuals, intended to promote welfare, similarly detailed standards designed to uphold animal welfare do not exist for circuses [6,20]. Due to the travelling nature of the industry, circuses provide conditions that are normally sub-optimal compared to those of zoos, with subsequent limitations often compromising each of the five welfare domains. This assumption is supported by Gupta and Chakraborty [25], who described the role of zoos in rehabilitating tigers sourced from circuses, given that the welfare conditions within zoos are usually substantially better. By making inferences from research conducted in zoos, where welfare is generally considered better, this study yields conclusions that are conservative.

### 1.3. Research Objectives

To date, published assessments of the welfare of tigers within circuses has considered only limited aspects or domains of welfare, and very limited numbers of relevant studies. The objective of this research was to conduct a thorough assessment of the welfare of tigers within circuses, considering all relevant peer-reviewed studies published until 2023, and all of the Five Domains.

## 2. Methods

Personal and potentially subjective opinions of experts are considered less reliable than more objective scientific literature analyses [26]. Narrative literature reviews often focus on a subset of research, based on availability or author choice. These can create conscious or unconscious biases during the selection and inclusion of scientific evidence [27]. By contrast, systematic literature reviews aim to minimise bias by identifying and analysing all relevant studies on a specific topic, using robust and transparent criteria. These are considered to provide evidence of the greatest level of reliability when exploring scientific topics, and their use for such purposes is considered best practice [26,27,28].

Systematic reviews require a detailed search strategy and defined inclusion and exclusion criteria prior to starting the review, all of which should be made transparent. The identification process often utilises electronic databases, but can also be supplemented by checking reference lists or manually searching key journals to promote completeness and reliability.

To assess scientific evidence as to the welfare of tigers in captivity, considered via the Five Domains approach, a systematic literature review was performed, following PRISMA guidelines [28]. The key aims of this research were to identify, evaluate, collate, and analyse all relevant peer reviewed scientific studies aimed at providing insights into the welfare impacts, both positive and negative, of keeping tigers in travelling circuses.

Web of Science and Scopus were used as the literature databases for the surveys. These were chosen as they are among the world’s most comprehensive life sciences databases, jointly providing a very high level of inclusion of all published scientific studies, holding over 155 million and 77.8 million records, respectively [29,30].

**Figure 1:**
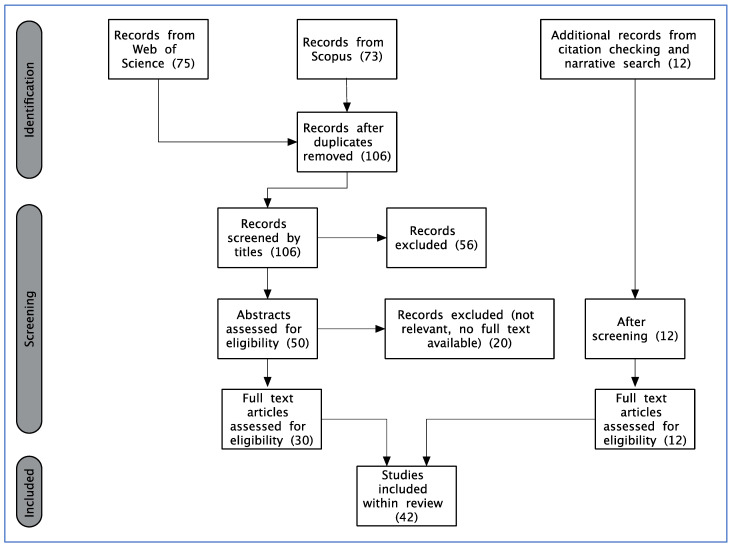
Literature review steps.

Both scientific literature surveys searched for terms within study titles, abstracts, and keywords. For Web of Science this was performed using ‘Topic’ search. Several search terms were used, with the aim of capturing the relevant studies which were combined into the following search string: TS (Topic) = (tiger OR *Panthera tigris* OR big cat OR felid) AND (circus OR captive OR captivity OR entertainment) AND (welfare OR five domains OR five freedoms). For Scopus the following search string was used: Article title, abstract or keywords = (tiger OR {*Panthera tigris*} OR {big cat} OR felid) AND (circus OR captive OR captivity OR entertainment) AND (welfare OR {five domains} OR {five freedoms}). The Web of Science search was conducted on 11 November 2022, and the Scopus search on 19 November 2022. There was no limit on the publication years considered. Duplicates of papers identified in both surveys were removed to provide a single list of unique references for review (Figure 1).

Studies that assessed or reviewed the welfare of tigers in captivity inclusive of management practices and the captive environment were defined as relevant. Therefore, any studies that did not specifically mention tigers, captivity, or welfare were excluded. Studies were first screened by title, and then by abstract. If uncertainty remained concerning study relevance, the full article was accessed to assess the main body of the text. Excluded from further analysis were studies without a full text in English available. For each of the relevant studies, data were extracted on the following parameters: (1) year of publication, (2) article type, (3) type of captive environment (e.g., circus), and (4) which welfare domains were reviewed or assessed (e.g., nutrition). All screening was conducted by one author to ensure consistency.

Despite the comprehensive nature of this scientific literature search, to minimise the risk of excluding any relevant studies, a narrative (i.e., non-systematic) search was also conducted for relevant papers within references of identified articles. Articles were screened using the same inclusion and exclusion criteria by the same independent reviewer.

## 3. Results

In total, 75 references were identified through Web of Science, and 73 through Scopus. Following removal of duplicates, this provided 106 unique references, of which 34 were relevant studies that assessed the welfare of tigers in captivity, and only three of which focussed specifically on circuses. Four papers were then excluded as no full text was available, leaving 30 studies to answer the research objective. The oldest paper identified in this systematic search was published in 1997, and the most recent in 2022, with 23 papers published during the most recent decade (from 2013–2022). A narrative search identified a further 12 papers, published between 1991 and 2015, including an additional five which focussed on the circus environment. The methods used to identify the final list of unique studies included within this analysis are summarised in Figure 1. The number of studies identified, screened, retained, or discarded at each stage are shown. Data for studies retained and included in the review, are summarised within Table 1.

## 4. Discussion

### 4.1. Nutrition

The nutrition domain assesses welfare based on the availability of food and water and the suitability of nutrition [24]. Whilst the availability of clean water is relatively easy to control within captivity, the difference between wild and captive diets is a major limitation of captive environments. Nutritional aspects such as dietary quality, quantity, and feeding frequency are subject to various degrees of management challenges. The difficulty in controlling these is heightened in travelling circuses due to the nature of moving locations and thus difficulty in sourcing quality meat or other ingredients. Problems in nutrition can lead to obesity, emaciation, diabetes, and other medical conditions, or in some cases, death [17,24].

In the wild, a Bengal tiger (*Panthera tigris tigris*) would have a varied diet consisting of deer species, such as Chital (*Axis axis*) and Sambar (*Rusa unicolor*), whereas diet in captivity usually consists of beef, chicken, horse, rabbit, or in rare cases, buffalo [24,32,45,58]. This difference in diet can result in nutritional deficiencies or microbiota imbalances that can cause severe health concerns, such as bone pathologies and gastrointestinal diseases [24,45]. The literature suggests that feeding beef and muscle meat may be related to gastrointestinal diseases, whereas the provision of skeletal components, such as long bones, which can aid dental health, may be beneficial [45]. However, captive tigers are most commonly fed commercial raw meat diets consisting of muscle meat.

A study by Veasey [63] explained that whilst the provision of daily meals helps in monitoring food consumption, this does not provide for the psychological needs of the animal, as it virtually eliminates naturally occurring behaviours like foraging. Not having the opportunity to perform such highly-motivated natural behaviours can be a cause of frustration and lead to expression of stereotypical behaviours, which are often associated with poor welfare [47]. Stereotypical behaviours, or stereotypies, are repetitive, often invariant behavioural patterns, with no obvious function or goal [47,65]. These behaviours are regularly used as an indicator of sub-optimal welfare and are associated with long-term stress or an inability to perform species-specific behaviours, indicating that the environment does not meet the individual’s needs.

Stereotypical behaviours are diverse and can vary by species and even individual, with common examples including pacing, bar gnawing, excessive grooming, and even self-harming [56,57,66]. Pacing is the most frequently exhibited stereotypy in captive felids. In a survey by Mason and Latham [67], 68% of the environments in which stereotypies were recorded were associated with diminished welfare. In some cases, stereotypies are correlated with perseveration and could therefore be learned [66]. They may also persist, even after welfare issues have been resolved; for example, an individual may continue to exhibit stereotypies that were established within a previous poor environment. Determining the cause or motivation behind an underlying stereotypy can therefore be difficult; however, it is generally considered that animals displaying stereotypies have been exposed to sub-optimal welfare and are experiencing chronic stress [50].

Feeding large carcasses can enable animals to express foraging behaviours; however, bones can sometimes present choking hazards, and this method of feeding can make it more difficult to monitor food consumption [63]. An autopsy of a captive Bengal tiger, published in 2022, found poultry bones in the stomach [39]. The study hypothesised that the tiger exhibited geophagy (dirt-eating) of the sand substrate from its captive environment to alleviate indigestion caused from eating a rotten poultry carcass, ultimately resulting in death. Feeding boxes, feeding poles, or mixed feeding methods and routines could provide a source of enrichment to satisfy foraging behaviours, whilst also ensuring the safety of the animal [34,42,44,60]. Such feeding methods can also provide health benefits, with tigers that use feeding poles recording a mean arthrosis score that is four times less than those who did not [44], which may have been associated with the stimulation of greater physical activity. However, compared to zoos, the circus environment makes the delivery of these techniques more challenging due to limitations in space, time, and equipment.

### 4.2. Physical Environment

The physical environment domain evaluates the atmospheric conditions, enclosure size, transport situation, and presence of enrichment in determining positive or negative welfare outcomes. Nevill et al. [52] studied the impact of temperature on tigers during transportation, concluding that tigers are tolerant to extremes of both heat and cold. However, Schmidt-Burbach et al. [57] reported that inadequate conditions in captivity cause suffering, and that improvement of husbandry conditions should be considered a priority. One of the greatest limitations for tigers in captivity is enclosure size [32,62]. Animals with larger territories in the wild tend to fare worse in captivity and are more vulnerable to welfare problems [35]. Wild tigers often occupy large territories, with a median home range area of 48.40 km^2^, which cannot normally be provided for in captivity [62]. Brando [18] noted that transport requirements and small sub-standard housing conditions, common in circuses, reduce the opportunity for species-natural behaviours to be expressed, such as walking, foraging, and exploring [62]. Tigers frequently exhibit stereotypies in captivity as a result of the frustration experienced at not being able to perform these highly-motivated behaviours [18,19,34,37,42,51,62]. Pacing behaviour is commonly used as an indicator of poor welfare [59].

#### 4.2.1. Enclosure Size and Complexity

The size of the enclosure negatively correlates with the expression of stereotypical pacing, meaning that the smaller the enclosure, the more pacing is exhibited [33]. However, Gomes et al. [4] suggested that quality of the enclosure, in terms of complexity, is more important than the size, as environmental enrichment can help to reduce indicators of diminished welfare, such as pacing. A study by Biolatti et al. [32] recorded that only 0.43% of the activity budget was pacing for the tigers studied in enriched enclosures. This contrasts with other studies by De Rouck et al. [36], Mohapatra et al. [51], and Vaz et al. [62], which reported stereotypical behaviours in the activity budget for up to 24%, 23%, and 12%, respectively. The animals studied by Biolatti et al. [32] were housed in naturalistic enclosures, with trees, water pools, logs, and elevated platforms, in line with features that international guidelines recommend for captive tiger enclosures [6]. This suggests that environmental enrichment could reduce expression of stereotypic behaviours and even improve welfare. This theory is supported by many studies within the scientific literature, with welfare recommendations including provision of a multisensory space, dense vegetation, a variety of pathways to enable individual choice, presentation of novel items, and even olfactory enrichment [31,34,56,60,62,63].

Nevill and Friend [21] indicated that, on average, circus animals spend 1–9% of the day performing or training. For the remaining time, the animal is confined in exercise pens. Whilst they found that time in the exercise pen was important for animal welfare, the exercise pens in circuses often do not meet the minimum zoo standards for outdoor enclosures [19]. Exercise pens are often significantly smaller (only 26.3% of the recommended size for a zoo) and barren, with no vegetation or choice of environment, providing limited environmental enrichment [18,19]. The pens also rarely provide a hide or shelter for animals to express freedom of choice and retreat. The scientific literature indicates that larger enclosures, environmental enrichment, and vegetation cover can reduce expression of stereotypical behaviours and even improve welfare [42,60,62]. However, the nature of a travelling circus severely limits any ability to provide an appropriate physical environment for tigers [19].

#### 4.2.2. Transportation

Travel may cause animals stress due to forced movement, human handling during loading and unloading, noise, cage motion, and confinement, sometimes without access to food and water [19]. Iossa et al. [19] analysed 153 European and North American travelling circuses to determine the average time animals are in one place before being re-exposed to travel. The mean length of stay per location was only 6.9 days. Nevill and Friend [22] suggested that circus tigers may become habituated to transportation; however, there are very few studies that assess the effect of transport on the welfare of tigers in a travelling circus, and no conclusive evidence of this [18,19]. Dembiec et al. [37] conducted a study on sanctuary tigers, identifying increased levels of the key stress hormone cortisol in tigers exposed to the transport environment for the first time. This increase in cortisol is indicative of physiological stress and was found to last for 9–12 days after exposure to transportation. With transportation occurring in the circus on average every 6.9 days [19], this suggests that physiological and psychological stress could be sustained by some individuals throughout the travelling year. However, in the same study, tigers who had travelled at least twice previously (*n* = 2) did not show a pronounced cortisol spike, suggesting that tigers may become desensitized or habituated to transportation [37]. This study was conducted on a very small sample of sanctuary tigers, but suggests more research is required to better understand the welfare impact of transportation for circus tigers specifically, who are routinely exposed to this environment.

### 4.3. Health

Proper health care is essential to ensuring the positive welfare of an animal and can be assessed through the presence or absence of disease, injury, or functional impairment under the health domain [24,39,62]. Sethi et al. [58] noted that veterinary care is often available onsite at zoos, enabling regular vaccinations, deworming, routine health checks, and sample testing. This improved veterinary care contributes to increased longevity in such captive tigers [46]. Continued access to veterinary care is vital in ageing tigers as we continue to encounter novel degenerative diseases. According to the European Board of Veterinary Specialisation (EBVS), there were only 73 veterinarians specialised in zoo health management for ‘exotic’ animals, such as tigers, across the world, by June 2023 [68]. Table 2 shows the top five countries by number of specialists, indicating that access to specialists in other countries, such as Spain, which has not yet enforced a ban on the use of tigers in the circus, is even more challenging. Whilst zoos are able to employ routine healthcare, the same access is unlikely to be readily available in a travelling circus environment.

The health domain is often influenced by husbandry and management practices, such as diet, housing, enrichment opportunities, and even human contact [24,39,41,61]. For example, nutrient deficiencies have been linked to the development of bone pathologies in captive carnivores [24], and a reduced variety of food has been shown to generate imbalances in bacterial microbiota, negatively impacting immunity and resulting in intestinal inflammation, which can present as Crohn’s disease and ulcerative colitis [24]. Furthermore, inadequate housing or enrichment can cause chronic stress, which can result in physiological concerns, such as supressed immune function [19]. The case study reported by Hernández-Aco et al. [39] highlights the importance of providing adequate opportunities to engage in natural, highly motivated behaviours to reduce the prevalence of stereotypies due to stress in captivity.

### 4.4. Behavioural Interactions

This welfare domain includes human-animal interactions, alongside species-specific social behaviours [1]. In captivity, tigers are exposed to human-animal interactions daily, via both keepers and visitors. These interactions may lead to human-animal relationships that can be positive, neutral or negative in nature [40].

#### 4.4.1. Training

Interacting with large carnivores such as big cats is often dangerous for both the keeper and the animal. Negative experiences can result in tiger attacks and animal euthanasia [54,61]. Training via negative reinforcement or punishment may be a cause of poor welfare, which could reinforce unnatural behaviours, remove animal choice and control, decrease maternal rearing, and cause aggression [19,61]. Hands on training or handling has also been determined to be risky for tiger welfare, as it can cause disease transmission between animals and humans, encourage inappropriate pet ownership, and is in general unnecessarily dangerous. The results from a survey of 86 keepers on their handling experience with tigers show caution against the use of hands-on training or handling—which is often used in circuses—due to the potential for disease transmission between animal and zookeeper, and physical dangers, which can ultimately lead to injury and in some cases animal euthanasia [61]. However, training through positive reinforcement, with a protective barrier, could be beneficial to animal welfare and even considered a form of enrichment [19,48,61,64]. Studies suggest that positive reinforcement training can facilitate better medical care and can improve the physical and mental well-being of captive felids [61].

Positive human-animal relationships can be built through regular exposure to consistent keepers with a positive attitude who are utilising the right training techniques [40,62]. However, the presence of other variables in creating this positive experience, such as environmental enrichment, are not well documented. To determine the welfare implications of training, it is important to understand the methods used and to assess the specific human-animal relationship on a case-by-case basis [40].

#### 4.4.2. Human Disruption

Loud noises and the presence of a human audience are known stressors for captive animals [40]. A circus environment, during a performance, could therefore be a significant stressor for tigers [18]. In a study by Krawczel et al. [23], stereotypical pacing peaked in the hours before performance. Whilst the study concluded that this was anticipatory, it could also be determined that this pacing was indicative of frustration or anxiety [24]. Conversely, in instances of environmental disruption, caused by construction around an enclosure, tigers have been shown to retreat rather than pace [38]. Retreat and hiding space is recommended for captive felids to help prevent the onset of stereotypes [62]; however, the exercise pens available to circus tigers rarely provide shelter into which tigers may retreat. Hence, pacing could instead result from such disruption, indicating stress and diminished welfare.

#### 4.4.3. Social Interactions

There are limited recommendations in the literature regarding the social housing of tigers, with studies revealing mixed findings. Szokalski et al. [60] suggested that, due to the behaviour of wild tigers, younger individuals would benefit from social housing more than adult tigers. Therefore, housing tigers with individuals of a similar age can be beneficial [62]. However, preference around sociality may also be impacted by individual tiger personality [55]. De Rouck et al. [36] recommended housing tigers in pairs, to decrease stress and associated stereotypic pacing behaviour. By contrast, a study by Miller and Kuhar [49] on a group of six female tigers reported an increase in non-contact aggression and a decrease in social proximity over a longer period. The literature is therefore inconsistent in terms of social housing recommendations.

In one study, the presence of neighbours was found to cause more stereotypical pacing—an indicator of increased stress and frustration—leading to the conclusion that the welfare of tigers with neighbouring conspecifics is lower than their counterparts without neighbours [36]. The inclusion of a visual barrier can help to reduce pacing [50]. Due to the limited space in circuses, social housing of tigers could be a welfare concern, particularly during transportation.

Cubs should remain with their mothers to provide nutritional, developmental, and behavioural benefits [43]. However, excessive noise, unsuitable birthing locations, or high levels of disturbance can necessitate hand rearing. Reintroduction thereafter should be gradual using scent trials and protective barriers.

### 4.5. Mental State

The four domains previously analysed (nutrition, physical environment, health, and behavioural interaction) are considered ‘physical’ [24]. However, the fifth domain assesses the mental state and subjective experience of an animal, which is strongly impacted by the other four domains. The mental state can be negative or positive and it is important to consider that state will differ between individuals based on their subjective experiences, which can also be affected by individual personality [2,4,5,55]. Negative experiences include pain, fear, distress, boredom, and frustration, whilst positive experiences include calmness, playfulness, and satiety [13]. Brando [18] notes that life in a circus could also impact on other desired behaviours that are not often considered for research in zoos or the wild; for example, an animal’s desire for a certain view, or for control over how long an activity lasts.

Behavioural time budgets, collected through use of an ethogram, are commonly used to assess the prevalence of behavioural indicators of mental state [32,59]. Psychological priorities of wild and captive tigers include foraging, walking, exploring, chasing prey, territorial marking, and exercising freedom of choice [60,63]. Expression of these behaviours can be considered as indicators of a positive mental state. Indicators of a negative mental state in tigers include stereotypical pacing, excessive grooming, aggression, fleeing, and other avoidance behaviours [32,34,56].

Whilst circuses can offer positive goal-directed rewards through training [61], there is clear evidence in the scientific literature of stereotypical pacing in tigers kept in captivity, including within circuses [18,19,34,37,42,51,53,57,62]. The literature also suggests that age and sex can impact on individual mental state. Middle aged tigers exhibit the greatest percentage of stereotypical behaviour, and male tigers exhibit greater pacing behaviour than females [33,62].

Similar levels of stereotypical pacing have been identified in both zoos (23%) and circuses (20%), indicating that both forms of captivity often provide a sub-optimal environment [37,53]. However, Biolatti et al. [32] recorded captive tiger activity budgets to show sleeping (32.64%), resting (27.50%), and walking (17.30%). Only 0.43% of the activity budget was attributed to the negative welfare indicator of pacing. Therefore, in large naturalistic enclosures, with features of environmental enrichment—as studied by Biolatti et al. [32]—tigers exhibit fewer behavioural indicators of poor welfare. This indicates that environmental enrichment and enclosure complexity is of key importance for captive tiger welfare, and this is difficult to achieve in a travelling circus environment.

## 5. Study Limitations

Across the literature review and supplementary narrative search, only eight studies specifically assessed the physiological and psychological welfare implications of tigers in circuses directly, indicating the need for additional research in this area. Further, most studies focussed on inputs of animal management, rather than individual animal-experienced outputs. Additional studies are required on circus tigers specifically, to confirm inferences of mental state. As research on the welfare of individuals in circuses is scarce, in accordance with methods in the literature, likely welfare implications were inferred from zoo data [19,24]. Given that circuses normally provide conditions that are sub-optimal compared to those of zoos due to the travelling nature of the industry, we expect welfare to be better in zoos and therefore, our results to be conversative. For example, the physical environment within circuses is often much smaller, with limitations on space and species-specific environmental enrichment, such as water pools and elevated platforms, which have been shown to improve welfare of tigers in permanent zoo enclosures. Further research could be conducted to assess welfare impacts holistically in the circus environment or specifically on the transportation and social housing of tigers. Future studies could also consider the geographic location of zoos or circuses where possible, as conditions will likely differ based on this.

In accordance with best practice, two databases were used for the systematic review. These were Web of Science and Scopus, which are among the largest in the health and life sciences, minimising the chance of missing any relevant studies [29,30]. However, further studies could expand the search across additional databases. The search term ‘Welfare’ was used to ensure that all retrieved studies were relevant to the research question. Despite these best practice research steps, there is a small chance that this search strategy could have excluded studies that reviewed various aspects of circus management without citing welfare as a key objective. Database results were screened by a single independent reviewer, to ensure consistency. However, screening could be conducted independently, by more individuals, to reduce the risk of bias. Further, studies without a full English language text available were excluded from review, meaning that other relevant studies published in alternative languages could also have been missed. However, the additional narrative search conducted based partly on article references can be expected to have located virtually all additional studies of significance. These steps provide strong confidence in the body of scientific evidence analysed, and the conclusions that may be inferred from it.

## 6. Summary of Conclusions

This study examined the welfare implications of keeping tigers in circuses through the Five Domains model, which is increasingly being used within animal welfare legal cases [14]. The scientific literature was comprehensively searched, with 42 relevant scientific studies examined. Collectively, these studies indicate that the travelling nature of a circus often negatively impacts the welfare domains of nutrition, physical environment, and health, predominantly due to difficulties in sourcing appropriate veterinary care or nutrition, and the limitations in size and complexity of temporary enclosures, and those suitable for regular transportation. With regards to behavioural interactions, the literature is divided concerning whether training can improve welfare through offering mental stimulation and reducing boredom. This is dependent on the training methods used. However, there were concerns over excessive disturbance and noise caused by human interactions, such as visitors. Tigers were shown to exhibit stereotypical behaviours in captivity, due to limited opportunity to engage in natural behaviours. A higher percentage of pacing—an indicator of stress and poor welfare—was recorded in the activity budget of animals housed in barren enclosures, and before circus performances. As such, the collective scientific evidence on this issue indicates that tigers are not well-suited to circus living, due to the inability of a travelling circus to provide for their species-specific psychological, physiological, and behavioural needs. Circuses frequently negatively impact the welfare of tigers, which is harmful to them.

Due to a lack of research conducted in circus environments, these evidence-based conclusions are mainly inferred from zoo research, although, by implication, welfare issues are more likely to apply in circuses. The absence of scientific research on animal welfare in the circus environment continues to impede the introduction of legislations or bans on circuses. However, this research supports additional nationwide bans on the use of tigers in travelling circuses internationally.

## Figures and Tables

**Table 1:** Key parameters and welfare domains addressed in the 42 unique studies identified through systematic and narrative literature reviews. The Five Domains are: (1) Nutrition, (2) physical environment, (3) health, (4) behavioural interactions, and (5) mental state.

Study	Article Type	Captive Environment	Welfare Domain				
			1	2	3	4	5
Antonenko et al., 2019 [31]	Original Article	Zoo		x			x
2. Biolatti et al., 2016 [32]	Original Article	Zoo	x	x			x
3. Brando, 2016 [18]	Book chapter	Circus		x		x	x
4. Breton and Barrot, 2014 [33]	Original Article	Zoo		x			x
5. Clayton and Shrock, 2020 [34]	Original Article	Zoo	x				x
6. Clubb and Mason, 2003 [35]	Communication	Zoo		x			x
7. De Rouck et al., 2005 [36]	Original Article	Zoo		x		x	x
8. Dembiec et al., 2004 [37]	Original Article	Sanctuary		x			x
9. Gomes et al., 2020 [4]	Original Article	Zoo		x			x
10. Harley et al., 2019 [38]	Original Article	Zoo				x	x
11. Hernández-Aco et al., 2022 [39]	Original Article	Zoo			x		x
12. Hosey and Melfi, 2015 [40]	Original Article	Zoo				x	x
13. Hussain et al., 2021 [41]	Original Article	Zoo			x		
14. Iossa et al., 2009 [19]	Review Article	Circus		x	x		x
15. Johnson and Langton, 2021 [42]	Original Article	Zoo	x				x
16. Kelling et al., 2013 [43]	Original Article	Zoo				x	
17. Krawczel et al., 2005 [23]	Original Article	Circus				x	x
18. Law and Kitchener, 2020 [44]	Review Article	Zoo	x		x		
19. Lefebvre et al., 2020 [45]	Original Article	Zoo	x		x		
20. Longley, 2011 [46]	Original Article	Zoo			x		
21. Mason, 1991 [47]	Review Article	Zoo/Laboratory					x
22. Mellen and Shepherdson, 1997 [48]	Original Article	Zoo	x	x			x
23. Miller and Kuhar, 2008 [49]	Original Article	Zoo				x	x
24. Miller et al., 2008 [50]	Original Article	Zoo				x	x
25. Mohapatra et al., 2014 [51]	Original Article	Zoo		x			x
26. Mota-Rojas et al., 2022 [24]	Review Article	Circus	x	x	x	x	x
27. Nevill and Friend, 2003 [22]	Original Article	Circus		x			x
28. Nevill and Friend, 2006 [21]	Original Article	Circus		x			x
29. Nevill et al., 2004 [52]	Original Article	Circus		x	x		
30. Nevill et al., 2010 [53]	Original Article	Circus		x			x
31. Nyhus et al., 2003 [54]	Original Article	Zoo/Private				x	
32. Pastorino et al., 2017 [55]	Original Article	Zoo				x	x
33. Rose et al., 2017 [56]	Original Article	Zoo		x			x
34. Schmidt-Burbach et al., 2015 [57]	Original Article	Zoo	x	x	x		x
35. Sethi et al., 2022 [58]	Original Article	Zoo	x		x		
36. Stanton et al., 2015 [59]	Original Article	Zoo					x
37. Szokalski et al., 2012 [60]	Review Article	Zoo	x	x		x	x
38. Szokalski et al., 2013 [61]	Original Article	Zoo			x	x	x
39. Vaz et al., 2017 [62]	Original Article	Zoo		x	x	x	x
40. Vaz et al., 2022 [5]	Review Article	Zoo			x	x	x
41. Veasey, 2020 [63]	Perspective	Zoo	x				x
42. Westlund, 2014 [64]	Review Article	Zoo				x	x

**Table 2:** Zoo Health Management specialists in the top 5 countries within the US, UK and EU, ordered by number of specialists, according to the EBVS as of 8 June 2023 [68,69,70,71,72,73].

Country	Number of Veterinarians	Number of Veterinarians Specialised in Zoo Health Management	% of Zoo Health Management Specialists
United States	124,069	19	0.02%
UK	14,771	18	0.12%
France	20,000	7	0.04%
Italy	31,040	4	0.02%
Switzerland	2100	4	0.19%

## Data Availability

The original contributions presented in the study are included in the article. Further inquiries can be directed to the corresponding author.

## References

[1] Mellor D.J., Beausoleil N.J., Littlewood K.E., McLean A.N., McGreevy P.D., Jones B., Wilkins C. (2020). The 2020 Five Domains model: Including human–animal interactions in assessments of animal welfare. Animals.

[2] Broom D., Knight A., Phillips C., Sparks P. (2023). Animal welfare concepts. Routledge Handbook of Animal Welfare.

[3] Broom D.M. (1988). The scientific assessment of animal welfare. Appl. Anim. Behav. Sci..

[4] Gomes D., McSweeney L., Santos M. (2020). Effects of environmental enrichment techniques on stereotypical behaviours of captive Sumatran tigers: A preliminary case study. J. Anim. Behav. Biometeorol..

[5] Vaz J., McElligott A.G., Narayan E. (2022). Linking the roles of personality and stress physiology for managing the welfare of captive big cats. Anim. Welf..

[6] (2016). AZA Tiger Species Survival Plan. Tiger Care Manual.

[7] Mellor D.J., Reid C.S.W., Baker R.M., Jenkin G., Mellor D.J. (1994). Concepts of animal well-being and predicting the impact of procedures on experimental animals. Improving the Well-Being of Animals in the Research Environment.

[8] Mellor D.J., Stafford K.J. (2001). Integrating practical, regulatory and ethical strategies for enhancing farm animal welfare. Aust. Vet. J..

[9] Mellor D.J. (2004). Comprehensive assessment of harms caused by experimental, teaching and testing procedures on live animals. Altern. Lab. Anim..

[10] Mellor D.J., Patterson-Kane E., Stafford K.J. (2009). Animal welfare, grading compromise and mitigating suffering. The Sciences of Animal Welfare.

[11] Mellor D.J. (2012). Affective states and the assessment of laboratory-induced animal welfare impacts. Altex Proc..

[12] Mellor D.J., Beausoleil N.J. (2015). Extending the ‘Five Domains’ model for animal welfare assessment to incorporate positive welfare states. Anim. Welf..

[13] Mellor D.J. (2017). Operational details of the Five Domains model and its key applications to the assessment and management of animal welfare. Animals.

[14] Ledger R.A., Mellor D.J. (2018). Forensic use of the Five Domains Model for assessing suffering in cases of animal cruelty. Animals.

[15] Webster J., Knight A., Phillips C., Sparks P. (2023). Animal welfare concepts. Routledge Handbook of Animal Welfare.

[16] Four Paws Australia (2023). Worldwide Bans on Wild Animals in Circuses. https://www.four-paws.org.au/campaigns-topics/topics/wild-animals.

[17] Dorning J., Harris S., Pickett H. (2016). The Welfare of Wild Animals in Travelling Circuses. https://www.ispca.ie/uploads/The_welfare_of_wild_animals_in_travelling_circuses.pdf.

[18] Brando S., Bovenkerk B., Keulartz J. (2016). Wild animals in entertainment. Animal Ethics in the Age of Humans.

[19] Iossa G., Soulsbury C.D., Harris S. (2009). Are wild animals suited to a travelling circus life. Anim. Welf..

[20] Radford M. (2007). Wild Animals in Travelling Circuses. https://www.yumpu.com/en/document/read/16949284/wild-animals-in-travelling-circuses-the-report-archive-defra.

[21] Nevill C.H., Friend T.H. (2006). A preliminary study on the effects of limited access to an exercise pen on stereotypic pacing in circus tigers. Appl. Anim. Behav. Sci..

[22] Nevill C.H., Friend T.H. (2003). The behavior of circus tigers during transport. Appl. Anim. Behav. Sci..

[23] Krawczel P.D., Friend T.H., Windom A. (2005). Stereotypic behavior of circus tigers: Effects of performance. Appl. Anim. Behav. Sci..

[24] Mota-Rojas D., Ghezzi M.D., Domínguez-Oliva A., De la Vega L.T., Boscato-Funes L., Torres-Bernal F., Mora-Medina P. (2022). Circus animal welfare: Analysis through a five-domain approach. J. Anim. Behav. Biometeorol..

[25] Gupta B.K., Chakraborty B. (2005). The role of zoos in the rehabilitation of animals in the circus. J. Appl. Anim. Welf. Sci..

[26] Miller D.W., Miller C. (2005). On evidence, medical and legal. J. Am. Physicians Surg..

[27] Uman L.S. (2011). Systematic reviews and meta-analyses. J. Can. Acad. Child Adolesc. Psychiatry.

[28] Page M.J., McKenzie J.E., Bossuyt P.M., Boutron I., Hoffmann T.C., Mulrow C.D., Shamseer L., Tetzlaff J.M., Akl E.A., Brennan S.E. (2021). The PRISMA 2020 statement: An updated guideline for reporting systematic reviews. Brit. Med. J..

[29] Clarivate (2022). Web of Science Platform. Web of Science Platform—Web of Science Group. https://clarivate.com/.

[30] Elsevier (2020). Scopus Content Coverage Guide. https://www.elsevier.cn/products/scopus/metrics/#3-citescore.

[31] Antonenko T.V., Matsyura A.V., Pysarev S.V. (2019). Influence of Cinnamon on the behavior of Amur Tiger (*Panthera tigris* altaica, Temminck, 1844) in Captivity. Ukr. J. Ecol..

[32] Biolatti C., Modesto P., Dezzutto D., Pera F., Tarantola M., Gennero M.S., Maurella C., Acutis P.L. (2016). Behavioural analysis of captive tigers (*Panthera tigris*): A water pool makes the difference. Appl. Anim. Behav. Sci..

[33] Breton G., Barrot S. (2014). Influence of enclosure size on the distances covered and paced by captive tigers (*Panthera tigris*). Appl. Anim. Behav. Sci..

[34] Clayton M., Shrock T. (2020). Making a Tiger’s Day: Free-operant assessment and environmental enrichment to improve the daily lives of captive Bengal tigers (*Panthera tigris tigris*). Behav. Anal. Pract..

[35] Clubb R., Mason G. (2003). Captivity effects on wide-ranging carnivores. Nature.

[36] De Rouck M., Kitchener A.C., Law G., Nelissen M. (2005). A comparative study of the influence of social housing conditions on the behaviour of captive tigers (*Panthera tigris*). Anim. Welf.-Potters Bar Then Wheathampstead.

[37] Dembiec D.P., Snider R.J., Zanella A.J. (2004). The effects of transport stress on tiger physiology and behavior. Zoo Biol. Publ. Affil. Am. Zoo Aquar. Assoc..

[38] Harley J.J. (2019). Effects of assembly and operation of an amusement ride on the behaviour of a pair of captive Amur tigers (*Panthera tigris* altaica). J. Zoo Aquar. Res..

[39] Hernández-Aco R.S., Villarroel M., Miranda-de la Lama G.C. (2022). Geophagia in a large felid in captivity: A case report of lethal gastrointestinal impaction in a Bengal tigress (*Panthera tigris tigris*). J. Vet. Behav..

[40] Hosey G., Melfi V. (2015). Are we ignoring neutral and negative human–animal relationships in zoos?. Zoo Biol..

[41] Hussain S., Bukhari S.M., Wang L., Khalid N., Hou Z. (2021). Exploration of Zoo felids in North-East China for the prevalence and molecular identification of *Cryptosporidium* spp. PeerJ.

[42] Johnson B., Langton J. (2021). Behaviour change in Amur tigers *Panthera tigris* altaica after an enclosure move. J. Zoo Aquar. Res..

[43] Kelling A.S., Bashaw M.J., Bloomsmith M.A., Maple T.L. (2013). Socialization of a single hand-reared tiger cub. J. Appl. Anim. Welf. Sci..

[44] Law G., Kitchener A.C. (2020). Twenty years of the tiger feeding pole: Review and recommendations. Int. Zoo Yearb..

[45] Lefebvre S.L., Wallett H.M., Dierenfeld E.S., Whitehouse-Tedd K.M. (2020). Feeding practices and other factors associated with faecal consistency and frequencies of vomiting and diarrhoea in captive tigers (*Panthera tigris*). J. Appl. Anim. Nutr..

[46] Longley L. (2011). A review of ageing studies in captive felids. Int. Zoo Yearb..

[47] Mason G.J. (1991). Stereotypies: A critical review. Anim. Behav..

[48] Mellen J.D., Shepherdson D.J. (1997). Environmental enrichment for felids: An integrated approach. Int. Zoo Yearb..

[49] Miller A., Kuhar C.W. (2008). Long-term monitoring of social behavior in a grouping of six female tigers (*Panthera tigris*). Zoo Biol. Publ. Affil. Am. Zoo Aquar. Assoc..

[50] Miller L.J., Bettinger T., Mellen J. (2008). The reduction of stereotypic pacing in tigers (*Panthera tigris*) by obstructing the view of neighbouring individuals. Anim. Welf..

[51] Mohapatra R.K., Panda S., Acharya U.R. (2014). Study on activity pattern and incidence of stereotypic behavior in captive tigers. J. Vet. Behav..

[52] Nevill C.H., Friend T.H., Toscano M.J. (2004). Survey of transport environments of circus tigers (*Panthera tigris*). J. Zoo Wildl. Med..

[53] Nevill C.H., Friend T.H., Windom A.G. (2010). An evaluation of exercise pen use by circus tigers (*Pathera tigris tigris*). J. Appl. Anim. Welf. Sci..

[54] Nyhus P.J., Tilson R.L., Tomlinson J.L. (2003). Dangerous animals in captivity: Ex situ tiger conflict and implications for private ownership of exotic animals. Zoo Biol. Publ. Affil. Am. Zoo Aquar. Assoc..

[55] Pastorino G.Q., Paini F., Williams C.L., Faustini M., Mazzola S.M. (2017). Personality and sociality in captive tigers (*Panthera tigris*). Annu. Res. Rev. Biol..

[56] Rose P.E., Nash S.M., Riley L.M. (2017). To pace or not to pace? A review of what abnormal repetitive behavior tells us about zoo animal management. J. Vet. Behav..

[57] Schmidt-Burbach J., Ronfot D., Srisangiam R. (2015). Asian elephant (*Elephas maximus*), pig-tailed macaque (*Macaca nemestrina*) and tiger (*Panthera tigris*) populations at tourism venues in Thailand and aspects of their welfare. PLoS ONE.

[58] Sethi N., Chauhan N.S., Singh D.N., Vashisth S. (2022). Conserving Big Cats in Captivity: Management practices used by National Zoological Park, New Delhi, India. J. Wildl. Biodivers..

[59] Stanton L.A., Sullivan M.S., Fazio J.M. (2015). A standardized ethogram for the felidae: A tool for behavioral researchers. Appl. Anim. Behav. Sci..

[60] Szokalski M.S., Litchfield C.A., Foster W.K. (2012). Enrichment for captive tigers (*Panthera tigris*): Current knowledge and future directions. Appl. Anim. Behav. Sci..

[61] Szokalski M.S., Litchfield C.A., Foster W.K. (2013). What can zookeepers tell us about interacting with big cats in captivity?. Zoo Biol..

[62] Vaz J., Narayan E.J., Dileep Kumar R., Thenmozhi K., Thiyagesan K., Baskaran N. (2017). Prevalence and determinants of stereotypic behaviours and physiological stress among tigers and leopards in Indian zoos. PLoS ONE.

[63] Veasey J.S. (2020). Can zoos ever be big enough for large wild animals? A review using an expert panel assessment of the psychological priorities of the amur tiger (*Panthera tigris* altaica) as a model species. Animals.

[64] Westlund K. (2014). Training is enrichment—And beyond. Appl. Anim. Behav. Sci..

[65] Manning A., Dawkins M.S. (2012). An Introduction to Animal Behaviour.

[66] Mason G., Clubb R., Latham N., Vickery S. (2007). Why and how should we use environmental enrichment to tackle stereotypic behaviour?. Appl. Anim. Behav. Sci..

[67] Mason G.J., Latham N. (2004). Can’t stop, won’t stop: Is stereotypy a reliable animal welfare indicator. Anim. Welf..

[68] European Board of Veterinary Specialisation, n.d Specialists. https://www.ebvs.eu/specialists.

[69] European Board of Veterinary Specialisation, n.d Countries: France. https://www.ebvs.eu/countries/france.

[70] European Board of Veterinary Specialisation, n.d Countries: Italy. https://www.ebvs.eu/countries/italy.

[71] European Board of Veterinary Specialisation, n.d Countries: Spain. https://www.ebvs.eu/countries/switzerland.

[72] European Board of Veterinary Specialisation, n.d Countries: United Kingdom. https://www.ebvs.eu/countries/united-kingdom.

[73] American Veterinary Medical Association (2023). U.S. Veterinarians. https://www.avma.org/resources-tools/reports-statistics/market-research-statistics-us-veterinarians.

